# Supplementary material for: Investigating the influence of physiologically relevant hydrostatic pressure on CHO cell batch culture
Source: Sci Rep. 2021 Jan 8;11:162. doi: 10.1038/s41598-020-80576-8 (PMC7794228; doi:10.1038/s41598-020-80576-8)
Supplement: Supplementary file 1 — Supplementary Information 1. [file 41598_2020_80576_MOESM1_ESM.zip › S1/IgG/Feb17 60 90 standard/Feb13 90mmHg.pdf]

Data File C:\Users\P...mStation\1\Data\TK_CHO_IgG 2019-02-17 14-04-01\004-3-90mmHg Feb13.D
Sample Name: 90mmHg Feb13

===

Acq. Operator : SYSTEM Seq. Line : 4
Sample Operator : SYSTEM
Acq. Instrument : HPLC-0039 Location : 3
Injection Date : 2/17/2019 2:17:56 PM Inj : 1
Inj Volume : 100.000 µl
Method : C:\Users\Public\Documents\ChemStation\1\Data\TK_CHO_IgG 2019-02-17 14-04-01\CHO_IgG_LC.M (Sequence Method)
Last changed : 2/8/2019 3:17:23 PM by SYSTEM

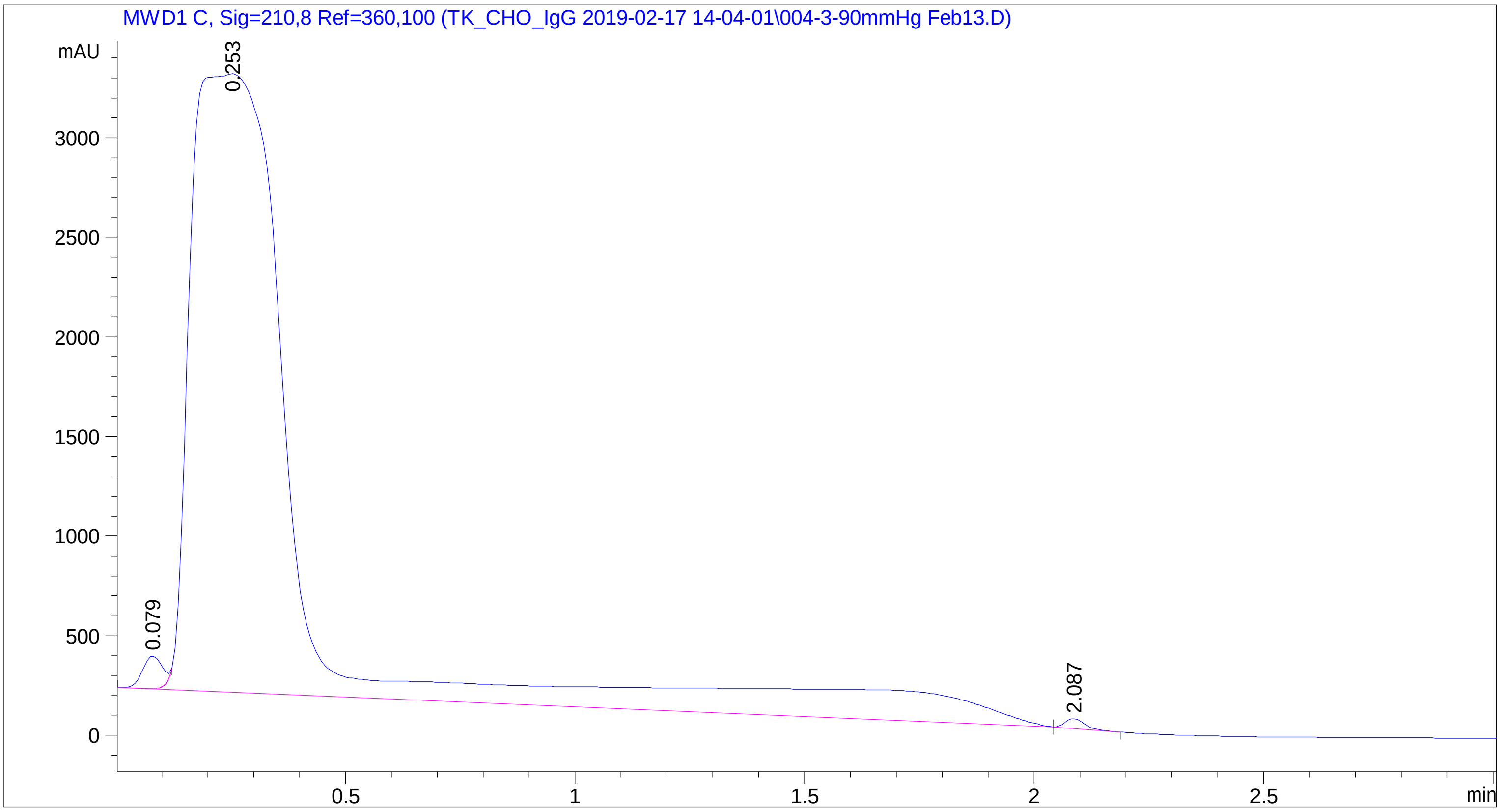

===

Area Percent Report

===

Sorted By : Signal
Multiplier : 1.0000
Dilution : 1.0000
Use Multiplier & Dilution Factor with ISTDs

Signal 1: MWD1 C, Sig=210,8 Ref=360,100

Peak #	RetTime [min]	Type	Width [min]	Area [mAU*s]	Height [mAU]	Area %
1	0.079	BV E	0.0466	481.54135	162.52493	0.9511
2	0.253	VB R	0.2238	5.00041e4	3106.83228	98.7605
3	2.087	BBA	0.0469	146.05992	48.85397	0.2885

Totals : 5.06317e4 3318.21118

===

*** End of Report ***
